# Supplementary material for: Essential Role of MHC II in the Antitubercular Efficacy of Pyrazinamide
Source: bioRxiv. 2025 Aug 21:2025.08.21.671522. Preprint. [Version 1] doi: 10.1101/2025.08.21.671522 (PMC12393548; doi:10.1101/2025.08.21.671522)
Supplement: 1 [file NIHPP2025.08.21.671522V1-supplement-1.pdf]

bioRxiv preprint doi: https://doi.org/10.1101/2025.08.21.671522; this version posted August 21, 2025. The copyright holder for this preprint (which was not certified by peer review) is the author/funder, who has granted bioRxiv a license to display the preprint in perpetuity. It is made available under aCC-BY 4.0 International license.

Supplementary Data.

Table S1: Log differences in statistically significant treatment comparisons in *Mtb* infected C57BL6/J mice BMDMs.

		**Day 4**	
**C57BL6/J**	**Comparison**	**Log$_{10}$ Difference**	***P* -value**
	PZA$_{400}$ v. ND	0.912	0.0278
	IFN-γ v. ND	1.51	0.0201
	IFN-γ + PZA$_{200}$ v. ND	2.11	0.0184
	IFN-γ + PZA$_{400}$ v. ND	2.77	0.0180
	PZA$_{400}$ v. PZA$_{200}$	0.782	0.035
	IFN-γ v. PZA$_{200}$	1.38	0.0223
	IFN-γ v. PZA$_{400}$	0.6	0.05
	IFN-γ + PZA$_{200}$ v. PZA$_{200}$	1.977	0.0199
	IFN-γ + PZA$_{400}$ v. PZA$_{200}$	2.64	0.0013
	IFN-γ + PZA$_{200}$ v. PZA$_{400}$	1.19	0.0187
	IFN-γ + PZA$_{400}$ v. PZA$_{400}$	1.86	0.0192
	IFN-γ + PZA$_{400}$ v. IFN-γ	1.26	0.0189
	IFN-γ + PZA$_{400}$ v. IFN-γ + PZA$_{200}$	0.663	0.05
		**Day 5**	
	**Comparison**	**Log$_{10}$ Difference**	***P* -value**
	PZA$_{400}$ v. ND	1.23	0.0079
	IFN-γ v. ND	2.22	0.0041
	IFN-γ + PZA$_{200}$ v. ND	3.02	0.0038
	IFN-γ + PZA$_{400}$ v. ND	3.60	0.0031
	PZA$_{400}$ v. PZA$_{200}$	0.868	0.0345
	IFN-γ v. PZA$_{200}$	1.85	0.0190
	IFN-γ v. PZA$_{400}$	0.984	0.0269
	IFN-γ + PZA$_{200}$ v. PZA$_{200}$	2.65	0.0178
	IFN-γ + PZA$_{400}$ v. PZA$_{200}$	3.24	0.0034
	IFN-γ + PZA$_{200}$ v. PZA$_{400}$	1.78	0.0188
	IFN-γ + PZA$_{400}$ v. PZA$_{400}$	2.37	0.0045
	IFN-γ + PZA$_{200}$ v. IFN-γ	0.8	0.0322
	IFN-γ + PZA$_{400}$ v. IFN-γ	1.38	0.0082
	IFN-γ + PZA$_{400}$ v. IFN-γ+ PZA$_{200}$	0.585	0.0356

bioRxiv preprint doi: https://doi.org/10.1101/2025.08.21.671522; this version posted August 21, 2025. The copyright holder for this preprint (which was not certified by peer review) is the author/funder, who has granted bioRxiv a license to display the preprint in perpetuity. It is made available under aCC-BY 4.0 International license.

Table S2: Log differences in statistically significant treatment comparisons in *Mtb* infected C57BL6/NJ mice BMDMs.

		**Day 4**	
**C57BL6/NJ**	**Comparison**	**Log$_{10}$ Difference**	***P* -value**
	PZA$_{400}$ v. ND	0.692	0.0394
	IFN-γ v. ND	1.04	0.0244
	IFN-γ + PZA$_{200}$ v. ND	1.33	0.0201
	IFN-γ + PZA$_{400}$ v. ND	1.85	0.0174
	PZA$_{400}$ v. PZA$_{200}$	0.360	0.0031
	IFN-γ v. PZA$_{200}$	0.709	0.0003
	IFN-γ + PZA$_{200}$ v. PZA$_{200}$	1	0.0003
	IFN-γ + PZA$_{400}$ v. PZA$_{200}$	1.54	0.0002
	IFN-γ + PZA$_{200}$ v. PZA$_{400}$	0.64	0.0461
	IFN-γ + PZA$_{400}$ v. PZA$_{400}$	1.18	0.0211
	IFN-γ + PZA$_{400}$ v. IFN-γ	0.831	0.0032
	IFN-γ + PZA$_{400}$ v. IFN-γ + PZA$_{200}$	0.54	0.0010
		**Day 5**	
	**Comparison**	**Log$_{10}$ Difference**	***P* -value**
	PZA$_{400}$ v. ND	0.953	0.0427
	IFN-γ v. ND	1.34	0.0322
	IFN-γ + PZA$_{200}$ v. ND	1.87	0.0282
	IFN-γ + PZA$_{400}$ v. ND	2.77	0.0269
	PZA$_{400}$ v. PZA$_{200}$	0.6	0.0077
	IFN-γ v. PZA$_{200}$	0.985	0.0034
	IFN-γ v. PZA$_{400}$	0.389	0.0003
	IFN-γ + PZA$_{200}$ v. PZA$_{200}$	1.51	0.0025
	IFN-γ + PZA$_{400}$ v. PZA$_{200}$	2.41	0.0022
	IFN-γ + PZA$_{200}$ v. PZA$_{400}$	0.914	<0.0001
	IFN-γ + PZA$_{400}$ v. PZA$_{400}$	1.82	<0.0001
	IFN-γ + PZA$_{200}$ v. IFN-γ	0.524	0.0090
	IFN-γ + PZA$_{400}$ v. IFN-γ	1.43	0.0033
